# Irisin as a Neuroprotective Agent in Parkinson’s Disease: The Role of Physical Exercise in Modulating Dopaminergic Neurons

**DOI:** 10.3390/pharmacy14010009

**Published:** 2026-01-13

**Authors:** José Garcia de Brito-Neto, Paulo Leonardo de Góis Morais, José Rodolfo Lopes de Paiva Cavalcanti, Francisco Irochima Pinheiro, Fausto Pierdoná Guzen, Ricardo Ney Cobucci

**Affiliations:** 1 Laboratory Experimental Neurology, Department of Biomedical Sciences, Faculty of Health Sciences, State University of Rio Grande do Norte (UERN), Mossoró 59607-360, Brazil; jgarcia.academico@hotmail.com (J.G.d.B.-N.); pauloleonardo87@hotmail.com (P.L.d.G.M.); rodolfolopes@uern.br (J.R.L.d.P.C.); faustoguzen@uern.br (F.P.G.); 2 Postgraduate Program in Physiological Sciences, Department of Biomedical Sciences, Faculty of Health Sciences, State University of Rio Grande do Norte (UERN), Mossoró 59607-360, Brazil; 3 Postgraduate Program in Health and Society, Department of Biomedical Sciences, Faculty of Health Sciences, State University of Rio Grande do Norte(UERN), Mossoró 59607-360, Brazil; 4 Postgraduate Program in Biotechnology, Health School, Potiguar University (UnP), Natal 59056-000, Brazil; irochima@gmail.com

**Keywords:** irisin, Parkinson’s disease, neuroprotection, physical exercise, dopaminergic neurons

## Abstract

Exercise-induced myokines have emerged as crucial mediators of the beneficial effects of physical activity on neurodegenerative diseases through complex molecular mechanisms involving oxidative stress reduction, neuroinflammation suppression, and synaptic plasticity enhancement. Among these myokines, irisin, encoded by the FNDC5 gene, has gained significant attention as a potential therapeutic target in neurodegenerative conditions due to its ability to cross the blood–brain barrier and exert pleiotropic neuroprotective effects. This review synthesizes current evidence from both preclinical and clinical studies examining the role of exercise-induced irisin in neurodegeneration, with particular emphasis on translational potential and therapeutic applications. A comprehensive search was conducted across PubMed, Web of Science, Scopus, and EMBASE databases (spanning January 2015 to December 2024) to identify peer-reviewed articles investigating irisin’s neuroprotective mechanisms in neurodegenerative diseases. Ten studies met the inclusion criteria (five rodent/primate model studies and five human clinical investigations), which were analyzed for methodological rigor, intervention protocols, biomarker quantification methods, and reported outcomes. Reviewed studies consistently demonstrated that exercise-induced endogenous irisin elevation correlates with improved cognitive function, reduced neuroinflammatory markers, enhanced synaptic plasticity, and modulation of neurodegenerative pathways, with exogenous irisin administration reproducing several neuroprotective benefits observed with exercise training in animal models. However, substantial heterogeneity exists regarding exercise prescription parameters (intensity, duration, frequency, modality), training-induced irisin quantification methodologies (ELISA versus mass spectrometry), and study designs (ranging from uncontrolled human observations to randomized controlled trials in animal models). Critical appraisal reveals that human studies lack adequate control for confounding variables including baseline physical fitness, comorbidities, concurrent medications, and potential sources of bias, while biochemical studies indicate distinct pharmacokinetics between endogenous training-induced irisin and exogenous bolus dosing, necessitating careful interpretation of therapeutic applicability. The translational potential of irisin as a therapeutic agent or drug target depends on resolving methodological standardization in biomarker measurement, conducting well-designed clinical trials with rigorous control for confounders, and integrating findings from molecular/biochemical studies to elucidate mechanisms linking irisin to disease modification. Future research should prioritize establishing clinical trial frameworks that harmonize exercise prescriptions, employ robust biomarker quantification (mass spectrometry), and stratify participants based on disease stage, comorbidities, and genetic predisposition to clarify irisin’s role as a potential therapeutic intervention in neurodegenerative disease management.

## 1. Introduction

Neurodegenerative diseases including Parkinson’s disease, Alzheimer’s disease, and other progressive neurological conditions represent a growing global health burden with limited disease-modifying therapeutic options [1]. Recent epidemiological evidence (2023–2025) continues to establish regular physical activity as one of the most robust non-pharmacological interventions for slowing cognitive decline and motor symptom progression in these conditions, with mechanisms extending far beyond simple neurophysiological stimulation. Physical exercise induces a spectrum of myokine production in skeletal muscle, which are soluble signaling molecules that exert endocrine effects on distant tissues including the central nervous system. The muscle–brain axis represents a dynamic communication network whereby exercise-induced metabolites and myokines, including irisin, lactate, cathepsin B, neuregulin-1, and others, cross the blood–brain barrier and modulate neuroprotective pathways involving trophic factor signaling, immune regulation, and metabolic homeostasis [2,3,4].

Irisin is a 12 kDa proteolytic cleavage product of fibronectin type III domain-containing protein 5 (FNDC5), an exercise-inducible transmembrane protein predominantly expressed in skeletal muscle. In response to endurance training, resistance exercise, and high-intensity interval training, muscular FNDC5 is upregulated via peroxisome proliferator-activated receptor gamma coactivator 1-alpha (PGC-1α) signaling, subsequently undergoing proteolytic cleavage to release soluble irisin into the circulation [2,5]. Circulating irisin concentrations in humans typically range from 0.3 to 4 ng/mL when measured by mass spectrometry, with exercise-induced increases ranging from 10 to 30% depending on training intensity and duration. Given irisin’s reported ability to cross the blood–brain barrier and engage neurotrophic pathways, particularly through upregulation of brain-derived neurotrophic factor (BDNF), suppression of pro-inflammatory cytokines, and enhancement of synaptic plasticity mechanisms, irisin represents a candidate therapeutic target for neurodegeneration [2,6,7].

However, significant controversies remain regarding irisin detection methodologies, with important implications for interpreting study findings. Earlier studies employed antibody-based methods, including Western blotting and ELISA, that have been demonstrated to have substantial specificity and cross-reactivity issues, whereas contemporary mass spectrometry (MS) approaches provide superior sensitivity and specificity for identifying true irisin peptide signatures (FIQEVNTTTR fragment) [3]. This technical distinction carries profound implications for comparing studies and assessing the strength of evidence for irisin’s therapeutic potential. Furthermore, the distinction between endogenous exercise-induced irisin elevation and exogenous bolus administration of recombinant irisin represents a critical pharmacological distinction that has received insufficient attention in the current literature, as these approaches entail fundamentally different pharmacokinetics and may exert divergent biological effects at the cellular and tissue levels [8,9,10].

Experimental work has robustly demonstrated that irisin activates intracellular cascades controlling autophagy, mitochondrial homeostasis, and inflammatory signaling in neural and non-neural tissues. In PD models using α-synuclein preformed fibrils or MPTP, exogenous irisin consistently reduces NLRP3 inflammasome activation, lowers IL-1β, IL-6 and TNF-α levels, restores antioxidant defenses, rescues mitochondrial membrane potential, and preserves dopaminergic neurons with concomitant improvement in motor behavior, indicating a causally supported, reproducible pathway from irisin to reduced oxidative stress, improved mitophagy, and neuroprotection [9,10].

The current review synthesizes evidence from both preclinical animal models and human clinical studies to critically evaluate irisin’s role in neurodegeneration, assess translational potential, and identify critical gaps necessitating future research. Rather than presenting a descriptive compilation of study findings, this review applies rigorous critical appraisal to interrogate methodological robustness, reconcile contradictory findings across biochemical and clinical domains, and propose evidence-based frameworks for designing future well-controlled clinical trials.

## 2. Materials and Methods

### 2.1. Search Strategy and Data Sources

A systematic literature search was conducted across four major medical databases: PubMed, Web of Science, Scopus, and EMBASE. The search strategy employed Medical Subject Headings (MeSH) and keyword combinations including (irisin OR FNDC5 OR “fibronectin type III domain-containing protein 5”) AND (exercise OR training OR “physical activity”) AND (neurodegeneration OR neuroprotection OR Parkinson OR Alzheimer OR dementia OR “cognitive decline” OR neuroinflammation). The search encompassed literature published between 1 January 2015 and 31 December 2024 to capture both foundational studies establishing irisin’s neuroprotective mechanisms and recent clinical investigations, with no language restrictions applied to initial screening. Retrieved articles were exported to a reference management system and deduplicated.

### 2.2. Inclusion and Exclusion Criteria

Inclusion criteria were (1) peer-reviewed original research articles (both preclinical animal model studies and human clinical trials); (2) primary investigation of irisin/FNDC5 in the context of exercise, physical activity, or training interventions; (3) examination of neuroprotective outcomes, mechanistic pathways, or biomarker correlations in neurodegenerative disease contexts or related animal models of neurodegeneration; (4) provision of quantitative data on irisin levels, FNDC5 expression, or related neuroprotective endpoints. Exclusion criteria encompassed review articles, commentaries, case reports, letters to editors, studies lacking original data, investigations focused exclusively on non-neurological applications of irisin (e.g., bone health, metabolic syndrome without neurological assessment), and articles not available in full-text format.

### 2.3. Study Selection and Data Extraction

Two independent reviewers screened titles and abstracts against predetermined inclusion/exclusion criteria, with disagreements resolved through consensus discussion or consultation with a third reviewer. Full-text review of potentially eligible articles was subsequently conducted using standardized screening forms, and data extraction was performed using a structured template capturing study design classification (randomized controlled trial, quasi-experimental, cross-sectional, preclinical animal model), sample characteristics (number of subjects, age, disease status, clinical diagnosis), intervention protocols (exercise type, intensity, duration, frequency, training progression), irisin/FNDC5 quantification methodology (antibody-based method [Western blot, ELISA] versus mass spectrometry approach), reported biomarker concentrations and biochemical/molecular outcomes, cognitive or motor endpoints, methodological quality assessment, and authors’ reported conclusions. For studies involving multiple intervention arms or follow-up periods, separate data extraction entries were completed to capture time-dependent changes.

### 2.4. Data Synthesis and Critical Appraisal Framework

Rather than limiting analysis to descriptive summary of study findings, this review employed a structured critical appraisal framework interrogating

1. Exercise Prescription Standardization: How have training regimens (intensity, duration, frequency, modality, progression schemes) differed across reviewed studies, and what standardized frameworks should guide future investigations to enable robust inter-study comparison?
2. Biomarker Quantification Methodology: Which irisin detection approaches (ELISA versus MS) were employed in each study, and how might methodological differences in specificity/sensitivity/cross-reactivity account for discrepancies in reported irisin concentrations and intervention responsiveness?
3. Endogenous versus Exogenous Distinction: Which studies examined exercise-induced endogenous irisin elevation versus exogenous bolus irisin administration, and how might fundamental pharmacokinetic differences between these approaches influence biological interpretation and therapeutic applicability?
4. Translational Mechanistic Integration: How do findings from biochemical and molecular studies (including intracellular signaling pathways, FNDC5 cleavage mechanisms, cellular uptake, and receptor engagement) inform interpretation of preclinical animal studies and human clinical outcomes?
5. Control for Confounding Variables in Human Studies: What was the adequacy of control for potential confounders including baseline physical fitness, comorbidities, concurrent medications, and potential sources of bias, and how might unmeasured or uncontrolled variables influence study conclusions?
6. Disease Stage and Heterogeneity: Across reviewed studies, how did disease severity, disease stage (prodromal versus symptomatic), disease duration, and participant heterogeneity regarding comorbidities and genetic predisposition influence intervention responsiveness and irisin production?
7. Clinical Trial Design Implications: What specific recommendations do reviewed studies provide regarding optimal clinical trial design parameters (control group selection, outcome measurement, sample size, intervention duration, follow-up period, stratification variables) for well-controlled future investigations?

## 3. Results

### Study Selection and Characteristics

The initial search retrieved 847 articles, duplicates were removed, and 312 underwent full-text review after title/abstract screening. Ten studies met final inclusion criteria (five preclinical animal model investigations and five human clinical studies), with articles excluded due to lack of original neuroprotection data in neurodegeneration context (*n* = 156), focus on non-neurological irisin applications (*n* = 89), review articles or commentaries without novel data (*n* = 48), and insufficient methodological detail or non-English/Portuguese language publications (*n* = 9). Key characteristics of included studies are presented in Table 1.

Studies included five pre-clinical investigations in rodent/primate models of PD and five human studies with cross-sectional, observational, or interventional designs. Eaton et al. [11] demonstrated that acute high-intensity interval training (HIIT) elicits robust FNDC5 mRNA upregulation in vastus lateralis muscle and corresponding elevation in circulating irisin concentrations measured by ELISA. The authors proposed this finding as mechanistic support for exercise-induced irisin as a myokine mediator of neuroprotection.

Kim and Kim’s [12] study represents the only identified human study with genuine control group comparison, randomly assigning PD patients to 12-week aquarobic exercise intervention (3×/week, 60 min) versus usual care control. Irisin levels (ELISA), BDNF (ELISA), and cognitive testing (MMSE) were measured at baseline and post intervention. Results demonstrated significant elevation in irisin and BDNF with corresponding cognitive improvements in exercise group versus controls.

Jóźków et al. [14] measured circulating irisin (ELISA) in 25 elite marathon runners before, immediately after, and 24 h post race. Paradoxical finding: Despite extreme exercise stimulus (marathon duration ~3–4 h), irisin concentrations decreased 30% immediately post race compared to baseline, with partial recovery by 24 h. Authors attributed decrease to acute inflammatory response and metabolic stress overwhelming myokine secretion.

Kam et al. [15] employed SH-SY5Y dopaminergic neuroblastoma cells overexpressing wild-type α-synuclein, treated with exogenous irisin (100–500 nM), and measured α-synuclein oligomerization (ELISA), phosphorylated α-synuclein (Western blot), proteasomal activity, autophagy markers (LC3-II/LC3-I ratio), and cell viability. Results demonstrated dose-dependent reduction in α-synuclein aggregation and enhanced autophagy with irisin treatment.

Missaglia et al. [16] measured irisin in both serum and saliva of healthy volunteers (*n* = 45) before, immediately after, and 1, 2, 6, and 24 h post acute exercise bout (30 min moderate intensity running). Results demonstrated peak serum irisin at 1 h post exercise with return to baseline by 6 h; salivary irisin showed parallel but attenuated elevations. Authors proposed salivary irisin as non-invasive biomarker for exercise-induced myokine response.

Tang et al. [17] employed MPTP (1-methyl-4-phenyl-1,2,3,6-tetrahydropyridine)-lesioned mice (standard chemically induced PD model) assigned to (1) sedentary control and (2) treadmill exercise (moderate intensity, 5 days/week, 8 weeks). Outcomes measured: Motor function (rotarod, pole test), cognitive function (Morris water maze), tyrosine hydroxylase (TH+) dopaminergic neuron count, striatal dopamine content, and neuroinflammatory markers (IL-6, TNF-α, IL-1β). The exercise group demonstrated significant improvements across all motor and cognitive endpoints, with reduced dopaminergic neuronal loss and normalized inflammatory markers.

Tung et al. [19] tested hypothesis that high-intensity interval training (HIIT) is superior to continuous moderate-intensity exercise in MPTP-lesioned mice. Surprisingly, moderate-intensity continuous exercise (40% VO2max, 60 min daily) produced superior motor recovery, greater dopaminergic neuron preservation (TH+ counts), and enhanced striatal BDNF expression compared to HIIT (80% VO2max, 2 min intervals).

Finally, a human cross-sectional study enrolled 95 PD patients (Hoehn and Yahr stages 1–4) and 50 age/sex-matched healthy controls [20]. Serum irisin quantified using LC-MS/MS (mass spectrometry gold standard), avoiding ELISA limitations. Physical activity assessed via validated questionnaire (International Physical Activity Questionnaire). Key findings: (1) PD patients demonstrated significantly lower circulating irisin compared to controls (mean 2.1 ± 0.8 ng/mL versus 3.9 ± 1.2 ng/mL, *p* < 0.001), (2) irisin inversely correlated with disease severity (Spearman r = −0.58, *p* < 0.001), (3) physically active PD patients showed higher irisin than sedentary counterparts but remained below control levels, and (4) irisin weakly correlated with UPDRS motor score (r = −0.42) and cognitive function (MoCA score, r = 0.35).

## 4. Discussion

The present review and critical appraisal of ten studies investigating irisin as a neuroprotective agent in Parkinson’s disease reveals a field characterized by intriguing mechanistic evidence combined with substantial methodological heterogeneity, unresolved paradoxes, and critical gaps limiting clinical translation. In humans, researchers have reported encouraging results, although the evidence is limited. The ten reviewed studies collectively provide evidence that (1) exercise elicits irisin elevation (though with unexplained paradoxes—Study 4), (2) irisin possesses in vitro neuroprotective properties (primarily at supraphysiologic concentrations), (3) preclinical animal models demonstrate motor/cognitive improvements with exercise or exogenous irisin, and (4) limited human observational data suggest associations between physical activity, irisin levels, and disease severity.

**Figure 1:**
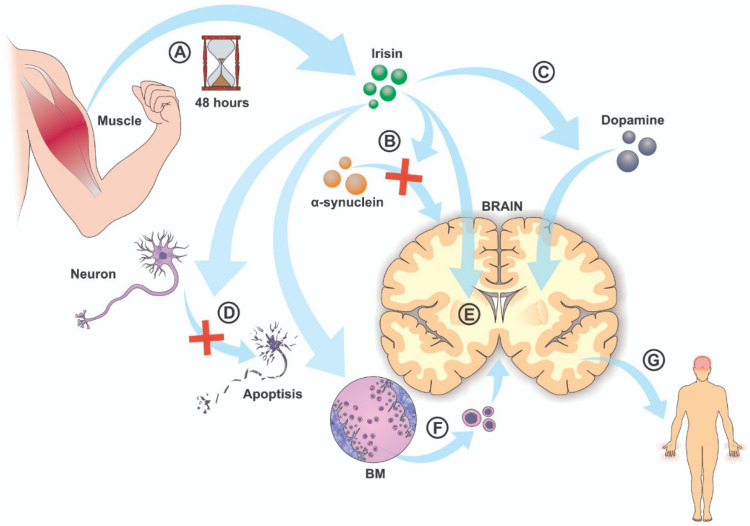
A—Physical exercise releases irisin. Its levels rise at the start of activity and stay high for 48 h. B—Irisin helps reduce the buildup of α-synuclein in the brain. C—Irisin increases dopamine uptake in the striatum. D—Irisin protects dopaminergic neurons. It lowers apoptosis, oxidative stress, and mitochondrial fragmentation while boosting mitochondrial respiration. E—Irisin promotes biogenesis. This leads to more dopaminergic neurons in the substantia nigra and striatum. F—Irisin helps stem cells move from the bone marrow to damaged brain areas. G—Irisin effects in the brain can ease Parkinson’s disease symptoms. This includes improving motor function, balance, and cognitive ability.

Current WHO and scientific society guidelines recommend neurodegenerative disease management: 150 min per week moderate-intensity aerobic activity plus two days weekly resistance/balance training (ACSM/AHA guidelines) [21]. Yet only a minority of reviewed human studies approached these targets (Kim and Kim achieved 180 min/week [12]; Zhang et al. achieved 135 min/week but short duration [18]). More critically, mechanisms linking specific exercise parameters (intensity thresholds, duration cumulation, frequency response, modality-specific effects) to irisin elevation remain unmapped. Figure 1 illustrates the release of irisin in the muscles during physical activity and its later effects.

Among the pathways depicted in Figure 1, the most robustly supported by experimental data are those related to α-synuclein clearance, mitochondrial protection, and dopaminergic neuron survival (panels B, C, D, and, indirectly, G). Exogenous irisin reproducibly reduces pathological α-synuclein species in cell culture and PFF-based mouse models, enhances lysosomal degradation, restores autophagy markers, and lowers NLRP3 inflammasome activation, with parallel reductions in oxidative stress and apoptosis and clear preservation of TH-positive nigrostriatal neurons and motor performance [22]. Similarly, there is strong mechanistic evidence that irisin improves mitochondrial function and biogenesis, decreasing ROS and mitochondrial fragmentation while increasing complex I activity, PGC-1/TFAM signaling, and mitophagy [22], which aligns with the “protection of dopaminergic neurons” and “promotion of biogenesis” components of panels D and E.

FNDC5 is the membrane precursor whose exercise-induced upregulation in muscle and the brain leads to proteolytic release of circulating irisin; thus, irisin represents the soluble effector form of FNDC5 signaling. In the central nervous system, irisin and FNDC5 form a functional axis upstream of BDNF, in which PGC-1–driven FNDC5 expression and subsequent irisin signaling increase BDNF transcription and TrkB-dependent activation of MAPK, PI3K, and PLC-pathways, thereby supporting neuronal survival, synaptic plasticity, and cognitive performance in both experimental PD and other neurodegenerative settings [23].

The paradoxical finding of Jóźków et al. [14], that exhaustive marathon running paradoxically suppresses rather than elevates irisin, raises unanswered questions: (1) Is the irisin response to exercise intensity dependent, with extreme supramaximal stress triggering different physiologic responses than moderate training? (2) Does the inflammatory cascade of extreme exercise suppress myokine secretion despite the greatest muscular stimulus? (3) Might ELISA artifacts compromise measurement at high inflammation states? (4) Could irisin clearance exceed production during acute stress? Without addressing these mechanistic questions, proposing irisin as a universal mediator of exercise benefits lacks empirical foundation.

ELISA-based irisin quantification is subject to documented limitations [24,25]: (1) cross-reactivity with FNDC5 precursor protein and potential FNDC5 cleavage intermediates; (2) lack of standardized antibodies with confirmed isoform specificity; (3) influence of sample handling, temperature, and storage on FNDC5 integrity; (4) potential for false-positive results in high-inflammatory states (uncertain mechanism); and (5) poor inter-assay standardization across commercial kits. These limitations are neither acknowledged nor addressed in most reviewed studies, introducing systematic bias of unknown magnitude.

Mass spectrometry-based approaches, specifically LC-MS/MS with isotope dilution quantification using stable-labeled irisin standards, provide substantially greater specificity, accuracy, and reproducibility [26]. Shi et al. [20] employed this approach and identified PD-associated irisin reduction and stage-dependent decline, findings with greater methodologic credibility. However, even mass spectrometry does not definitively establish biologic activity: measured irisin concentrations represent circulating peptide, not necessarily bioavailable irisin capable of receptor binding, blood–brain barrier penetration, and downstream signaling activation. Post-translational modifications (phosphorylation, glycosylation, proteolytic processing), protein–protein interactions, and clearance kinetics influence functional bioavailability beyond total concentration.

A fundamental distinction inadequately addressed across reviewed literature concerns profound pharmacokinetic differences between endogenous irisin elevation through exercise training versus exogenous bolus irisin administration. Studies by Kam et al. [15] and Zhang et al. [18] employed exogenous irisin at concentrations far exceeding those achieved through physiologic exercise training. This distinction carries profound mechanistic implications [2]: (1) supraphysiologic exogenous irisin may activate integrin αVβ5 receptors with different kinetics and extent than physiologic endogenous elevation, (2) saturation of receptor-mediated uptake mechanisms may not occur with endogenous irisin but does with bolus dosing, (3) supraphysiologic irisin may activate off-target receptors or signaling cascades not engaged at physiologic concentrations, (4) exogenous irisin cannot recapitulate temporal dynamics and tissue distribution of endogenous myokine response, (5) clearance kinetics, cellular internalization, and subcellular localization likely differ between endogenous and exogenous irisin.

FNDC5 is expressed not only in skeletal muscle but also in bone, adipose tissue, brain, and other tissues [27]. Systemic upregulation of FNDC5/irisin could trigger pleiotropic effects beyond neuroprotection: effects on bone metabolism, adipose tissue biology, vascular function, immune regulation, and metabolic homeostasis. These off-target effects remain largely uncharacterized. Individual variation in FNDC5 promoter polymorphisms, integrin αVβ5 coding variants, and downstream signaling component genetic variation could substantially influence irisin response to exercise and therapeutic response to FNDC5-augmenting interventions [28]. However, no reviewed study examined pharmacogenomic factors.

Shi et al. [20] demonstrate irisin reduction in PD patients, inversely correlated with disease severity. These paradoxes, far from being limitations of current knowledge, represent genuine scientific opportunities. Rather than accepting surface-level explanations, focused mechanistic investigations directly addressing these paradoxes could advance understanding substantially [29].

Critical deficiencies fundamentally limit translation to clinical application: heterogeneity across studies precludes synthesis; endogenous and exogenous irisin may represent distinct pharmacologic entities; causal mechanisms remain speculative; confounding variables inadequately controlled in human studies; and most studies measure disparate endpoints preventing mechanistic integration. Reviewed studies demonstrate exceptional scientific interest in irisin but fall short of rigorous critical appraisal standards.

## 5. Conclusions

Research in animal models indicates that irisin, whether introduced externally or generated through exercise, provides large protection to dopaminergic neurons, improves motor function, and promotes synaptic plasticity. This includes the preservation of dopaminergic neurons, a reduction in oxidative stress, and improvements in mitochondrial function and synaptic connectivity. Studies involving humans also suggest that irisin may enhance motor function, cognitive performance, and dopamine regulation in individuals with Parkinson’s disease.

The ultimate question regarding irisin in PD is not whether irisin is “important” or “interesting” (clearly, the evidence suggests mechanistic plausibility), but rather whether irisin is therapeutically actionable, whether enhancing irisin production or signaling becomes disease-modifying in human PD. This question remains genuinely unanswered and warrants focused, rigorous investigation. Until such evidence materializes, enthusiasm regarding irisin as a therapeutic target should be tempered by recognition of substantial scientific uncertainties and methodological limitations currently constraining this field.

Future research should aim to clarify the mechanisms by which irisin protects neurons and identify optimal exercise regimens to maximize its production and benefits for Parkinson’s disease. Well-structured clinical trials are essential to confirm these findings and investigate irisin as a potential adjunct therapy for Parkinson’s disease. Additionally, researchers should examine how variables such as age, disease stage, and genetic differences influence the effectiveness of exercise-induced irisin production in individuals with Parkinson’s disease. By addressing these areas, future studies can strengthen the rationale for exercise- and irisin-based interventions in managing Parkinson’s disease.

## Figures and Tables

**Table 1:** Characteristics of the selected studies.

Study ID	Authors and Ref.	Year	Sample Type	Number of Subjects	Clinical Trial Type/Model	Intervention	Exercise Protocol	Irisin Measurement	Conclusion
Study 1	Eaton et al. [11]	2018	Humans	*n* = 20	Intervention study	High-intensity interval training	20 days HIIT	FNDC5 mRNA expression	~5-fold increase in FNDC5 mRNA at rest
Study 2	Kim et al. [12]	2018	Humans	*n* = 40 (elderly women)	Randomized controlled trial	Aquarobic exercise	16 weeks, 3×/week	ELISA (irisin, BDNF)	Higher irisin (*p* < 0.001) and BDNF (*p* < 0.05)
Study 3	Zarbakhsh et al. [13]	2019	Mice	*n* = variable	Preclinical (Parkinson’s model)	BMSC + irisin	Combined treatment	Tyrosine hydroxylase + neurons	Increased dopaminergic neurons; improved behavior
Study 4	Jóźków et al. [14]	2019	Humans	*n* = 50 (marathon)	Observational intervention	Marathon running	Single 42 km event	ELISA (serum irisin)	30% decrease post-marathon; 32% lower at 7 days
Study 5	Kam et al. [15]	2022	Mice	*n* = variable	Preclinical (Parkinson’s model)	Irisin injection	Exogenous administration	α-synuclein, dopamine neurons	Reduced α-synuclein; preserved dopamine neurons
Study 6	Missaglia et al. [16]	2023	Humans	*n* = 30	Intervention with time-course	Maximal exercise test	Cycling ergometer test	Serum/salivary irisin	Peak at 24 h; baseline at 48 h post-exercise
Study 7	Tang et al. [17]	2023	Mice	*n* = variable	Preclinical (Parkinson’s model)	Treadmill exercise	5 days/week, 30–60 min	Motor, cognition, BDNF	Alleviated motor dysfunction and cognitive impairment
Study 8	Zhang et al. [18]	2023	Humans + Mice	*n* = variable	Combined study (Parkinson’s)	Exercise (humans) + irisin injection (mice)	Humans: 12 weeks; Mice: injection	Motor function, irisin, mitochondria	Improved motor function; neuroprotection
Study 9	Tung et al. [19]	2024	Rats	*n* = variable	Preclinical (Parkinson’s model)	3 exercise types (treadmill low/high, swimming)	30 min/day, 10 weeks	Mitochondrial function, BDNF	Low-intensity treadmill most effective
Study 10	Shi et al. [20]	2024	Humans	*n* = variable	Cross-sectional observational	Routine physical activity	Self-reported activity	MS for irisin, motor/cognitive assessment	Higher irisin = better motor/cognitive function

Abbreviations: RCT = Randomized Controlled Trial; HIIT = High-Intensity Interval Training; ELISA = Enzyme-Linked Immunosorbent Assay; MS = Mass Spectrometry; BDNF = Brain-Derived Neurotrophic Factor; BMSC = Bone Marrow Stem Cells; Aβ = Amyloid-Beta; FNDC5 = Fibronectin Type III Domain-Containing Protein 5.

## Data Availability

No new data were created or analyzed in this study. Data sharing is not applicable to this article.
